# A Morphometric Analysis of Palatal Rugae Patterns in a Saudi Arabian Population

**DOI:** 10.7759/cureus.33058

**Published:** 2022-12-28

**Authors:** Abdulsalam Alshammari, Fathima Fazrina Farook, Lulu Alyahya, Maha Alharbi, Norah N Alazaz, Lubna AlKadi, Farraj Albalawi, Ali Aboalela

**Affiliations:** 1 College of Dentistry, King Saud Bin Abdulaziz University for Health Sciences, Riyadh, SAU; 2 Ministry of National Guard for Health Affairs, Dental Hospital, Riyadh, SAU; 3 Research and Development, King Abdullah International Medical Research Center, Riyadh, SAU

**Keywords:** forensic dentistry, rugae patterns, palatascopy, age identification, sex identification, palatal rugae

## Abstract

Background

Palatal rugae patterns are useful in the field of forensic dentistry. Ethnicity has a significant effect on the development and final morphological pattern of palatal rugae. This study focused on a morphological analysis of the palatal rugae in a Saudi population to determine if any differences based on age and gender could support identifying an individual.

Materials and methods

This cross-sectional study conducted at the College of Dentistry, King Saud bin Abdul Aziz University for Health Sciences, was undertaken to evaluate 496 dental casts from the participant database of Saudi nationals from Riyadh. The rugae were delineated using a sharp graphite pencil under adequate light and magnification. The rugae patterns were classified based on the length, shape, and direction of the rugae by two observers as per Thomas and Kotze's criteria.

Results

The asymptotic chi-square McNemar test indicated bilateral symmetry for all the characteristics of the palatal rugae, except for the backward and forward directions of the rugae. Two-way analysis of variance (ANOVA) revealed a statistically significant interaction between the effects of age group and gender on the primary rugae count (F(3, 488) = 7.466, p <0.05)). In addition, age had a statistically significant effect on the fragmentary rugae (p <0.05), and gender had a statistically significant effect on the circular and backward patterns of the rugae (p<0.05). The females had a higher incidence of backward-directed rugae and the males had more circular rugae. No other significant difference was evident, based on gender. The logistic regression analyses showed a significant association between the circular (OR=1.298; 95% CI= 1.061-1.588) and backward (OR= 0.898; 95% CI= 0.828-0.975) palatal rugae and gender. Also, there was a significant association of the fragmentary palatal rugae (PR) (OR=1.274; 95% CI= 1.084-1.498) with the age group younger than 16 years.

Conclusion

In a Saudi Arabian ethnic group, the varying type of length of the palatal rugae patterns can be used to identify the age group while the direction and shape can be used to determine gender, although with limited accuracy. Post-mortem identification may benefit from using them along with other reliable forensic tools. There is a need to conduct continued research on diverse populations and ethnic groups in order to evaluate the PR potential in forensic dentistry.

## Introduction

Forensic dentistry involves the medico-legal identification of patients via dental findings [1]. The identification of a deceased person requires a careful evaluation of dental records, DNA tests, fingerprinting, and other forms of post-mortem evidence. Forensic odontology mainly uses human dentition for identification purposes due to its resistance to external factors and internal changes. The palatal rugae have been used in forensic investigations, particularly in cases when the most frequently used methods of identification such as human teeth, visual identification, DNA matching, and fingerprints were unavailable or unclear.

Palatal rugae are anatomical folds located on the anterior third of the palate behind the incisive papillae. They are also known as “Plica palatine” and the study of these patterns to identify a person's identity is called palatoscopy or palatal rugoscopy [2,3]. The stability of palatal rugae, similar to fingerprints, makes it a relevant source of human identification. Palatal rugae are unique to each individual and no two patterns are identical in terms of characteristics, including orientation, length, shape, and position. Rugae can withstand decomposition changes for seven days after death [3] and rarely change in shape with age. They reappear after trauma or surgery and are protected by the lips, cheeks, tongue, buccal pad of fat, and teeth in case of fire or forceful trauma [4]. The design and structure of palatal rugae are not altered by chemicals, heat, disease, or trauma, and if they are destroyed, they are reproduced exactly at the same location [4].

The pattern of palatal rugae may change due to certain events, such as finger sucking in childhood, orthodontic treatment, extraction of adjacent teeth, and surgical palatal repair [4-6]. According to a recent systematic review, the visual matching accuracy was >90% despite significant changes in the dimensions of the palatal rugae pre- and post-treatment, indicating that morphology may have played an important role in the visual matching [7].

Studies characterizing the morphological patterns of palatal rugae in different populations have been performed [8-11]. The findings indicate that ethnicity may have a significant effect on the development and final morphological pattern of palatal rugae. Additionally, palatine rugae exhibit racial and gender differences [8,12]. There has been a limited number of rugoscopy studies conducted in Saudi Arabia and they mainly focus on gender and the influence of orthodontic treatment on the rugae patterns [13-17].

Only a few studies have looked into the influence of age on palatal rugae [18-20]. It was the purpose of this study to explore the patterns of palatal rugae in a Saudi population and to examine the differences based on age that could aid in identifying an individual.

## Materials and methods

This cross-sectional study was conducted at the College of Dentistry, King Saud Bin Abdulaziz University for Health Sciences, during 2021-2022, evaluating 496 dental casts from the participant database. All were Saudi nationals from the region of Riyadh. Only the dental casts with the patient’s age and gender, as well as dental and medical histories, were included in this study. We followed the Strengthening the Reporting of Observational Studies in Epidemiology (STROBE) checklist for cross-sectional studies. Prior to the dental treatment, consent was obtained from the participants, parents, or legal guardians for the use of the casts and investigations for research purposes.

A sample of 496 dental casts, divided into four age groups, was chosen to achieve a power of 0.90. This power assumed an F test used with a significance level of 0.050 and an effect size of 0.17. The group proportions were equal. The age groups (124 in each group) were: Age group A: 6 to 16 years (Child); Age group B: 17 to 30 years (Young adults); Age group C: 31 to 40 years (Middle-aged adults); Age group D: 41 years and above (Older age adults).

The sample size was calculated using PASS 2020, v20.0.4 (PASS 2020 Power Analysis and Sample Size Software (2020). NCSS, LLC. Kaysville, Utah, USA) [21].

Every cast received a serial number as well as the gender and age of the patient. The study protocol was approved by the King Abdullah International Medical Research Committee (NRC21R/246/06).

Eligibility criteria included full maxillary dentition (except for third molars), and the anterior third of the palate was free of air bubbles and voids. For accurate odontometric measurements, the following exclusion criteria were used: records with a history of the disease, trauma, or surgery of the palate or tuberosity, severe palatal congenital malocclusion disorders or asymmetries, any dental appliances in the upper teeth, previous orthodontic treatment, medication that would affect the periodontal soft tissue, and malposition or malalignment of the posterior maxillary teeth. 

The outlines of the rugae were traced using a microtip graphite pencil and examined for different patterns under adequate light and magnified using a hand lens (RS Pro magnifying glass, 5x Magnification) to enhance the visibility of the palatal rugae and examined macroscopically. The study casts were placed on a horizontal base and each stone cast was evaluated bilaterally. All the measurements were taken by two independent observers (NA & LA). Any doubts were clarified with a third observer (FF). Subsequently, palatal rugae were classified according to the classification of Thomas et al. [22]. This classification is composed of three components (Table 1). The first part classifies palatal rugae according to their length. The second part classifies palatal rugae according to their orientation. The direction of the rugae was determined by measuring the angle between the line joining its origin and termination and the line perpendicular to the median raphe. The third part classifies palatal rugae according to their shape into one of six major types: straight, curved, wavy, circular, diverging, and converging.

**Table 1:** Thomas and Kotze (1983) classification of palatal rugae Source: [22]

Length	Primary	More than 5 mm
	Secondary rugae	3 to 5 mm
	Fragmentary	Less than 3 mm
Orientation:	Forward	Forwardly directed rugae are associated with positive angles.
	Backward	Backwardly directed rugae are associated with negative angles.
	Perpendicular	Angle of zero degrees.
Shape	Straight	Run directly from their origin to termination.
	Curved	Have a simple crescent shape that is curved gently.
	Wavy	Curved rugae have a bend at the origin or termination.
	Circular	Display a definite continuous ring formation.
	Diverging	Rugae have the same origin medially and split laterally
	Converging	Rugae begin from more than one origin but they unite at their lateral portions.

**Figure 1:**
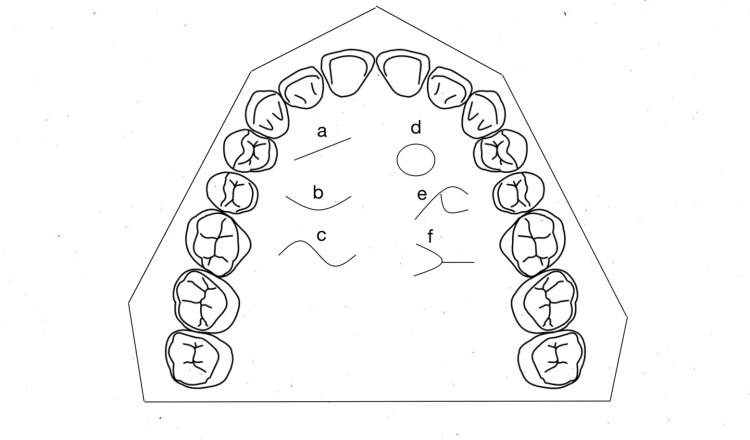
Recording parameters of the shape of rugae on the palate: (a) Straight, (b) Curved, (c) Wavy, (d) Circular, (e) Diverging, (f) Converging

**Figure 2:**
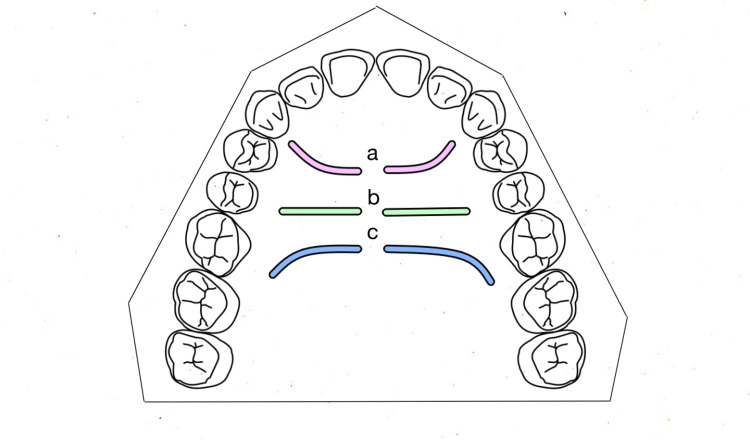
Direction: (a) Forward, (b) Perpendicular, (c) Backward

The data were entered into a Microsoft Excel spreadsheet (Microsoft Corporation, Redmond, WA) and analyzed. The shapes of palatal rugae are depicted in Figure 1 and the direction in Figure 2.

Statistical analysis

The analysis was performed using the statistical software NCSS version 2020 (NCSS, LLC, East Kaysville, Utah) [23]. Normality was assessed using the Kolmogorov-Smirnov test. The data were normal and parametric tests were conducted. More than two groups were evaluated by one-way or two-way analysis of variance (ANOVA) (in the case of two independent variables age and gender), followed by a post hoc analysis to evaluate the effect of the independent variables on the various palatal rugae patterns. Binary logistic regression analysis was performed to assess the gender and age group with significant palatal rugae patterns. Hosmer Lemeshow's goodness of fit was used to evaluate the model fit. A P-value of <0.05 was considered statistically significant.

## Results

A total of 496 casts were evaluated, 124 in each age group. The majority were female (n=270) and 226 males. Table 2 displays the distribution of the palatal rugae patterns by age group.

**Table 2:** Distribution of palatal rugae in different age groups

Rugae characteristics	Age group A			Age group B			Age group C			Age group D			Total
	R	L	Total N (%)	R	L	Total N (%)	R	L	Total N (%)	R	L	Total N (%)	
Length													
Primary	453	454	907 (24.63)	449	476	925 (25.12)	469	478	947 (25.72)	444	459	903 (24.52)	3682
Secondary	66	61	127 (25.53)	65	73	138 (27.77)	63	68	131 (26.36)	46	55	101 (20.32)	497
Fragmentary	49	62	111 (36.39)	34	26	60 (19.67)	42	22	64 (20.98)	41	29	70 (22.95)	305
Direction													
Backward	260	134	394 (28.59)	226	113	339 (24.60)	235	120	355 (25.76)	205	85	290 (21.04)	1378
Forward	112	241	353 (21.49)	145	282	427 (25.98)	158	279	437 (26.59)	146	280	426 (25.93)	1643
Perpendicular	182	195	377 (27.08)	172	165	337 (24.21)	178	172	350 (25.14)	173	155	328 (23.56)	1392
Shape													
Straight	102	98	200 (24.60)	90	85	175 (21.53)	124	101	225 (27.68)	89	124	213 (26.19)	813
Curved	126	138	264 (25.66)	116	122	238 (23.13)	116	124	240 (23.32)	152	135	287 (27.89)	1029
Wavy	165	160	325 (24.57)	171	166	337 (25.47)	167	197	364 (27.51)	152	145	297 (22.45)	1323
Circular	34	23	57 (23.85)	26	26	52 (21.76)	43	30	73 (30.54)	26	31	57 (23.85)	239
Diverge	59	65	124 (29.11)	51	47	98 (23.00)	61	47	108 (25.35)	50	46	96 (22.54)	426
Converge	11	9	20 (18.35)	10	18	28 (25.68)	17	10	27 (24.77)	15	19	34 (31.19)	109

In total, 2221 palatal rugae were on the right side, slightly less than on the left side (n=2263). The 6 to 16-year age group had more rugae (n=1145) compared to the other groups of age, followed by group C (n=1142), group B (n=1123), and the older group (n=1074). The most frequent type of rugae in all the groups was the wavy type, followed by the curved and straight types.

Diverging palatal rugae had a significantly higher incidence compared with the converging rugae. Regarding the length, the primary rugae were the predominant rugae, with the most frequent direction forward.

The current study detected no statistically significant difference between the right and left side values of all the variables. The asymptotic chi-square McNemar tests indicated a bilateral symmetry for all the characteristics of the palatal rugae (PR), except for the backward and forward directions of the rugae, which were not the same on both sides.

A two-way ANOVA was performed to analyze the effect of age and gender on the various characteristics of rugae (Table 3).

**Table 3:** Effects of gender and age on TAS scores: two-way ANOVA Note: *Indicates P≤0.05. PR, palatal rugae; SD, standard deviation, G, gender, Age grp, age group, SS, sum-of-squares, MS, mean squares, DF, degrees of freedom, TAS, Toronto Alexithymia Scale, Anova, analysis of variance

Rugae characteristics	Scores in each age group				SS	MS (DF)	F value	P-value
	Age group A	Age group B	Age group C	Age group D				
	Mean± SD (Median)	Mean± SD (Median)	Mean± SD (Median)	Mean± SD (Median)				
Length								
Total rugae	9.23±2.58 (9)	9.06±2.16 (9)	9.21±2.27(9)	8.66±2.65 (8)				
Age grp					32.352	10.784(3)	1.855	.136
Gender					14.247	14.247(1)	2.451	.118
G*Age					45.161	15.054(3)	2.590	.052
Primary	7.31± 1.68 (7)	7.46 ±1.61 (8)	7.64 ±1.85 (8)	7.28 ±1.63 (7)				
Age grp					11.022	3.674(3)	1.329	.264
Gender					00.091	.091(1)	.033	.856
G*Age					61.928	20.643(3)	7.466	.000*
Secondary	1.02±1.32 (1)	1.11± 1.07 (1)	1.06 ±1.37 (1)	0.81±1.17 (0)				
Age grp					7.912	2.637 (3)	1.729	.160
Gender					4.602	4.602 (1)	3.017	.083
G*Age					3.578	1.193 (3)	.782	.505
Fragmentary	0.89±1.53 (0)	0.48±0.89 (0)	0.52±1.04 (0)	0.57±1.08 (0)				
Age grp					16.247	5.416 (3)	4.017	.008*
Gender					1.761	1.761 (1)	1.306	.254
G*Age					4.342	1.447 (3)	1.073	.360
Direction								
Backward	3.18±2.36 (3)	2.73±2.21 (2)	2.87±2.26 (2)	2.34±2.19 (2)				
Age grp					33.275	11.092(3)	2.190	.088
Gender					23.419	23.419(1)	4.625	.032*
G*Age					9.837	3.279(3)	.648	.585
Forward	2.85±2.08 (3)	3.44±2.20 (3)	3.52±2.26 (3)	3.43±2.34 (3)				
Age grp					32.972	10.991(3)	2.225	.084
Gender					17.778	17.778(1)	3.599	.058
G*Age					3.883	1.294(3)	.262	.853
Perpendicular	3.04±2.82 (2.5)	2.72±2.16 (3)	2.82±2.36 (3)	2.65±2.43 (2)				
Age grp					16.959	5.653 (3)	.936	.423
Gender					4.747	4.747(1)	.786	.376
G*Age					18.438	6.146(3)	1.018	.385
Shape								
Straight	1.61±1.59 (1)	1.41±1.37 (1)	1.81±1.63 (1)	1.72±1.70 (1)				
Age grp					8.478	2.826 (3)	1.141	.332
Gender					.042	.042 (1)	.017	.897
G*Age					14.818	4.939(3)	1.995	.114
Curved	2.13±2.003 (2)	1.92±1.93 (2)	1.94±1.83 (2)	2.31±2.09 (2)				
Age grp					13.606	4.535 (3)	1.169	.321
Gender					1.542	1.542 (1)	.397	.529
G*Age					10.494	3.498 (3)	.902	.440
Wavy	2.62±1.93 (2.5)	2.72±2.03 (3)	2.94±2.06 (3)	2.39±1.80 (2)				
Age grp					19.278	6.426(3)	1.671	.172
Gender					1.971	1.971(1)	.513	.474
G*Age					8.332	2.777(3)	.722	.539
Circular	0.46±0.99 (0)	0.42±0.90 (0)	0.59±0.95 (0)	0.46±0.91 (0)				
Age grp					2.594	.865 (3)	.991	.397
Gender					6.522	6.522(1)	7.475	.006
G*Age					1.495	.498(3)	.571	.634
Diverge	1.0±0.954 (1)	0.79±0.85 (1)	0.87±0.91 (1)	0.77±0.90 (1)				
Age grp					4.494	1.498(3)	1.833	.140
Gender					.048	.048(1)	.059	.808
G*Age					3.367	1.122(3)	1.373	.250
Converge	0.16±0.41 (0)	0.23±0.46 (0)	0.22±0.45 (0)	0.27±0.52 (0)				
Age grp					.909	.303(3)	1.424	.235
Gender					.217	.217(1)	1.018	.314
G*Age					.134	.045(3)	.210	.889

**Figure 3:**
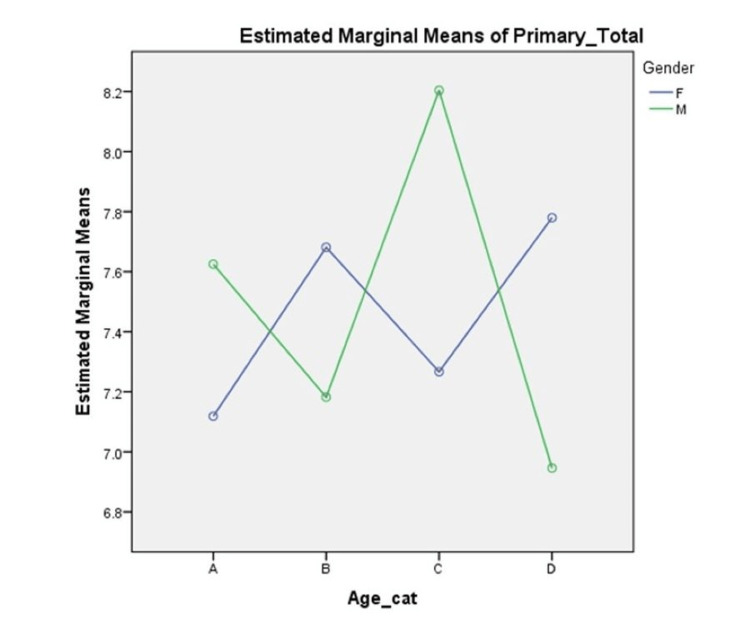
Plot of the estimated marginal means in primary rugae between genders in each age category

The two-way ANOVA revealed that there was a statistically significant interaction between the effects of age group and gender on the primary rugae counts (F(3, 488) = 7.466, p <0.05)). A graphical illustration of the interaction effect is displayed in Figure 3. The interaction effect of age group and gender on the primary rugae numbers is a set of non-parallel lines with the lines crossing at multiple places. The statistically significant interaction is evident as a complicated interaction, where the effects cannot be separated.

A simple main effects analysis showed that age has a statistically significant effect on fragmentary rugae (p <0.05). The post hoc tests associated with the age group variable for the fragmentary palatal rugae revealed that the P-value associated with the comparison between Group A and Group B in the Age variable is statistically significant (p = .028). The other group comparisons were not statistically significant. Based on the post hoc tests, the significant effect of age on fragmentary rugae was driven by Group A.

The simple main effects analysis showed that gender has a statistically significant effect on the circular and backward patterns of the rugae (p <0.05). The results indicated that the females had a higher incidence of backward-directed rugae (3.02 ± 2.316) compared to the males (2.49 ± 2.183); however, the males had a higher incidence of circular rugae (0.381 ± 0.751) compared to the females 90.602 ± 1.112). No other significant difference in the patterns was evident between the males and females.

The two independent binary logistic regression analyses to assess gender used significant variables in the bivariate analysis. There was a significant association of gender with the circular (OR=1.298; 95% CI= 1.061-1.588) and backward (OR= 0.898; 95% CI= 0.828-0.975) palatal rugae patterns (Table 4).

**Table 4:** Assessment of gender and age using different palatal rugae patterns Note: *Indicates P≤0.05. Abbreviations: OR, odds ratio; CI, confidence interval.

Dependent variable	Predictor variable Palatal Rugae Pattern	P-value	OR	95% CI
Gender (Ref Female)	Backward	0.0107*	0.898	0.828-0.975
	Circular	0.0112*	1.298	1.061-1.588
Age (Ref >16 years)	Fragmentary	0.028*	1.274	1.084-1.498

The same process was repeated for the age variable. There was a significant association between age and fragmentary palatal rugae (OR=1.274; 95% CI= 1.084-1.498), having fragmentary rugae increased the chances of predicting the age group 16 or less by a factor of 1.274.

## Discussion

This is the first study in Saudi Arabia to investigate the differences in the morphology of palatal rugae in terms of age. Another predictor was gender. We found a statistically significant effect for the length of the palatal rugae, specifically the fragmentary rugae between the age groups. The shape and direction did not vary with age. Only the backward-directed rugae and circular shape rugae were significantly different between males and females.

The distinctive characteristics of palatal rugae are permanent and stable and can be used as a method of establishing identity without antemortem records [24]. There have been numerous applications and facts about rugae in forensic dentistry documented in the literature. The individuality and accuracy of the patterns extend to monozygotic and dizygotic twins [11]. Additionally, rugae can be used for forensic identification in the absence of fingerprints. The fact that rugae patterns may differ among ethnicities is also highly beneficial. Kapali and colleagues examined the length, form, orientation, and unification of the palatal rugae in Australian Aborigines and Caucasians [25]. Numerous studies have demonstrated a substantial correlation between the various characteristics of rugae and ethnicity [26-34].

Our study evaluated the possibility of predicting age and gender using palatal rugae patterns, given the potential applications of palatoscopy in forensic dentistry. In our study, wavy and curved palatal rugae were most commonly reported, similar to other published studies [26-31]. The most frequent type of rugae in our study was forwardly directed. The backward and perpendicular type rugae were more frequent in the right half of the hard palate, and the forwardly directed rugae occurred more in the left half of the hard palate. The bilateral difference between palatal rugae types according to direction was statistically significant (P<0.001).

Age

Of the palatal rugae characteristics, the shape and direction of the rugae did not show any variation between the four age groups. When the different lengths of the rugae were compared by age groups, the fragmentary rugae were statistically significant (p <0.05). Although the simple main effect analysis did not show a significant effect between age and the primary rugae, there was a statistically significant interaction in the effects between age group and gender on the count of the primary rugae. Secondary types did not differ between the age groups. The findings support but differ slightly from other studies reporting a significant difference between the age groups and the total number of rugae [20]. We found that the total number of fragmentary rugae was less in the older age groups. The highest proportion of fragmentary rugae was found in the younger age group. The older age groups were more equal. As explained by Lysell, this could be due to the fact that the number of rugae, irrespective of the length type, increases during the development and remains the same until the age of 23 years, after which it declines [32].

There seems to be a slight difference between our findings and those of a similar study in which the number of all length types was reported to be lower in older individuals [20]. The number of palatal rugae was significantly different between patients in the younger age group and the group older than 41 years (P<0.003) [20]. It should be noted that the total number of participants in the older age group was very small in their study.

The number and shape of the palatal rugae were investigated and did not show any variations with the age groups. This finding disagrees with literature reporting significant differences in the direction of the rugae and shape between age groups [18,19].

We aimed to evaluate the role of palatal rugae in the assessment of age using logistic regression analysis. A direct comparison of the current study with existing studies may not be feasible, as only a limited number of studies are available. For every increase in fragmentary palatal rugae, the odds of being 16 or less years increase by a factor of 1.274 (OR=1.274; 95% CI= 1.084-1.498).

Gender

The gender differences in the Saudi population become more obvious in the orientation of the palatal rugae and circular shape. This study found that only the backward-directed rugae and circular shape rugae were significantly different by gender. The backward-directed rugae were significantly higher in females when compared with the males, which is similar to a previous study [27] and in opposition to the findings of Dwivedi et al. that reported that backward-directed rugae were frequent in males [33].

The current study found that the number of circular rugae was significantly higher in males, which is contradictory to findings reported in India [27,34]. However, another study reported the least occurring rugae pattern was circular in the females [20].

We observed no significant differences in the convergence and divergence patterns in males and females, similar to the literature [27,31,34]. However, literature also showed that males had more converging rugae, and females had more diverging rugae [35,36].

The stability and reliability of palatal rugae provide a reliable application in forensic identification. The ease of visualization and simplicity to record with minimal cost are some of the advantages of using this tool.

Limitations

The morphological changes over time in the palatal rugae were not analyzed and longitudinal studies should be performed to determine if significant differences are observed in the palatal area due to changes that the palatal mucosa endures over time. In light of the possible regional variations that can occur in Saudi Arabia, multicenter studies may be warranted. It would also be useful to measure the change in direction based on the angular changes recorded rather than classifying rugae in general terms. Finally, a long-term study would be beneficial to determine if the hard palate and rugae models are associated with developmental changes.

## Conclusions

Within the limitations of this study, we can conclude that in the palatal rugae patterns in a Saudi Arabian ethnic group, the type of length of palatal rugae have a possible role in identifying age. It can be used in conjunction with other reliable forensic tools as an additional tool for identifying gender. Further research is needed to examine palatal rugae's potential in forensic dentistry among diverse populations and ethnic groups. Our findings may shed light on the identification process of forensic anthropologists.
